# A Multilevel Perspective on the Skin: Skin Architecture, Microbial Communities, Intrinsic Metabolites, and Emerging AI-Enabled Applications

**DOI:** 10.3390/biology15211961

**Published:** 2026-11-01

**Authors:** Peikun Ma, Haihua Ruan, Hongyang Zhang

**Affiliations:** Tianjin Key Laboratory of Food Biotechnology, School of Biotechnology and Food Science, Tianjin University of Commerce, Tianjin 300134, China; pkd7328@163.com (P.M.); zhyang@tjcu.edu.cn (H.Z.)

**Keywords:** skin microbiome, skin metabolites, host–microbe interactions, microecological homeostasis, artificial intelligence

## Abstract

The skin is the largest organ of the human body and harbors a diverse microbial community that constitutes a complex ecosystem. Despite advances in next-generation sequencing (NGS) that have greatly facilitated the characterization of skin microbiota composition, a critical gap remains in understanding how intrinsic metabolites mediate host–microbe interactions and maintain microecological homeostasis. This review synthesizes current knowledge across interconnected layers of the skin ecosystem, including architecture, microbial communities, metabolites, and computational analytics. The skin’s structural features create spatially heterogeneous microenvironments that dictate microbial colonization patterns, while microbial communities have evolved adaptive strategies to exploit distinct metabolic niches. Intrinsic metabolites, encompassing lipids, proteins/peptides, ions, vitamins, and carbohydrates, constitute the chemical language through which host and microbes reciprocally communicate. The complexity of these interactions poses challenges for traditional analytical approaches, yet artificial intelligence is transforming the field by enabling progressive deepening of understanding, from predicting microbial functional potential to decoding the skin microbiome as a carrier of biological information. This multilevel framework provides a foundation for future investigations and may inform the development of personalized strategies for skin health management.

## Simple Summary

Skin is not just a barrier—it is a living ecosystem where human cells coexist with a diverse community of microorganisms, including bacteria, fungi, and viruses, as well as microscopic mites. Host skin cells form the physical and chemical foundation of this ecosystem, while the resident microorganisms and mites contribute to skin homeostasis by metabolizing skin-derived compounds, defending against pathogens, and modulating immune responses. However, when the balance among these components is disturbed, skin problems can occur. In addition, scientists still do not fully understand how the chemicals produced by our skin and by these microorganisms interact to maintain this balance. Recent advances in data analysis and artificial intelligence are now helping researchers study these complex interactions in new ways. This review brings together current knowledge on how skin structure, microorganisms, and skin-produced chemicals work together. By understanding these relationships, future research may be able to predict skin problems earlier and develop more personalized ways to keep skin healthy.

## 1. Introduction

The human skin is the largest organ of the human body and serves as the primary protective barrier between the internal environment and external insults. However, the skin is no longer viewed merely as a passive physical shield, but rather as a dynamic and complex ecosystem shaped by continuous host–microbe interactions. The surface of the skin hosts a specialized ecosystem colonized by millions of microorganisms including bacteria, fungi, viruses, archaea, and microeukaryotes, whose composition and function are dynamically influenced by local physicochemical conditions and host immunity [1,2]. These microorganisms do not merely reside on the skin surface; they engage in intimate and sustained associations with the host. Symbiosis has been defined as an association between two dissimilar organisms involving some degree of physical association that may persist over time, irrespective of its effects on the fitness of either organism [3,4]. Through these symbiotic relationships, microorganisms contribute to skin homeostasis by regulating the metabolism and transformation of host-derived compounds, maintaining skin barrier integrity, providing colonization resistance against potential pathogens, and modulating host immunity [5]. Despite these recognized contributions, the specific microbial composition and functional state that define a balanced or healthy skin microbiome remain poorly understood. The compositional ratio that defines microecological balance has yet to be identified. In fact, microbial community composition is highly personalized and temporally dynamic, varying across individuals and fluctuating throughout the lifespan [6]. This inherent variability poses a significant challenge for developing universally applicable diagnostic or therapeutic strategies.

Microbiota colonization begins at birth and is influenced by delivery mode (e.g., vaginal v.s. cesarean). Subsequently, skin microbiota composition is shaped by a combination of intrinsic factors (such as skin site, intra- and interpersonal variability, nutrient and space competition, ethnicity, gender, and age) and extrinsic factors (such as lifestyle, hygiene practices, cosmetic use, antibiotic exposure, geographical location, climate, and seasonality) [7]. Over the past two decades, advances in next-generation sequencing (NGS) technologies have greatly facilitated the characterization of skin microbiota composition. The Human Microbiome Project Consortium (HMP Consortium) found that the diversity and abundance of signature microbes vary widely among healthy subjects across different habitats [8,9]. Despite this diversity, a large-scale study from the National Institutes of Health (NIH) Roadmap Human Microbiome Project (Roadmap HMP) involving 242 phenotyped healthy adults revealed that the skin microbiota is predominantly composed of a small group of genera, *Staphylococcus*, *Cutibacterium*, and *Corynebacterium*, among others [10], which collectively accounted for more than 62% of sequences [11]. However, cataloging the microbial “who” and “where” represents only the first step. Considerable individual variability in skin microbiome composition persists, and a constructive benchmark for defining a balanced skin microecological state has not yet been established. This lack of systematic and longitudinal studies impedes our understanding of how microbial communities respond to external stimuli and how transient versus resident microbes contribute to overall ecosystem stability. Importantly, a critical gap remains in our understanding of how intrinsic metabolites provide the substance basis for skin microecology. Specifically, it remains unclear how the skin’s chemical microenvironment shapes microbial community assembly, and how microbial metabolism in turn remodels the host’s metabolic landscape. This reciprocal dialogue, mediated by metabolites, is only beginning to be unraveled. Compounding this complexity is the sheer volume and heterogeneity of data generated by modern multi-omics approaches. Traditional analytical methods are often limited in their capacity to integrate these high-dimensional datasets and extract biologically meaningful patterns that govern the ecosystem’s behavior. This bottleneck has catalyzed the emergence of artificial intelligence (AI) and machine learning as indispensable tools for decoding skin microecology. Although previous reviews have extensively summarized the skin microbiome and, separately, the metabolic landscape of the skin, comparatively less attention has been given to integrating skin architecture, microbial communities, and metabolites within a unified framework of skin microecology. In particular, the reciprocal relationships linking local skin microenvironments, microbial metabolic activities, and host physiology, as well as the potential role of AI in resolving these cross-level interactions, remain insufficiently integrated. Bridging these traditionally separated dimensions therefore represents an important step toward a more mechanistic understanding of skin microecological homeostasis. Previous research has established an integrative framework linking the skin microbiome, metabolome, and phenome, providing a comprehensive perspective on the interplay among microbial communities, metabolic processes, and host phenotypes [12]. Conversely, recent reviews on AI in dermatology have focused primarily on clinical applications (e.g., skin cancer diagnosis) rather than on the mechanistic integration of microbiome and metabolome data. The present review bridges these gaps by explicitly positioning metabolites as the chemical language connecting skin architecture and microbial communities, and by critically evaluating AI approaches through the lens of their capacity to decode this metabolite-mediated dialogue.

**Figure 1:**
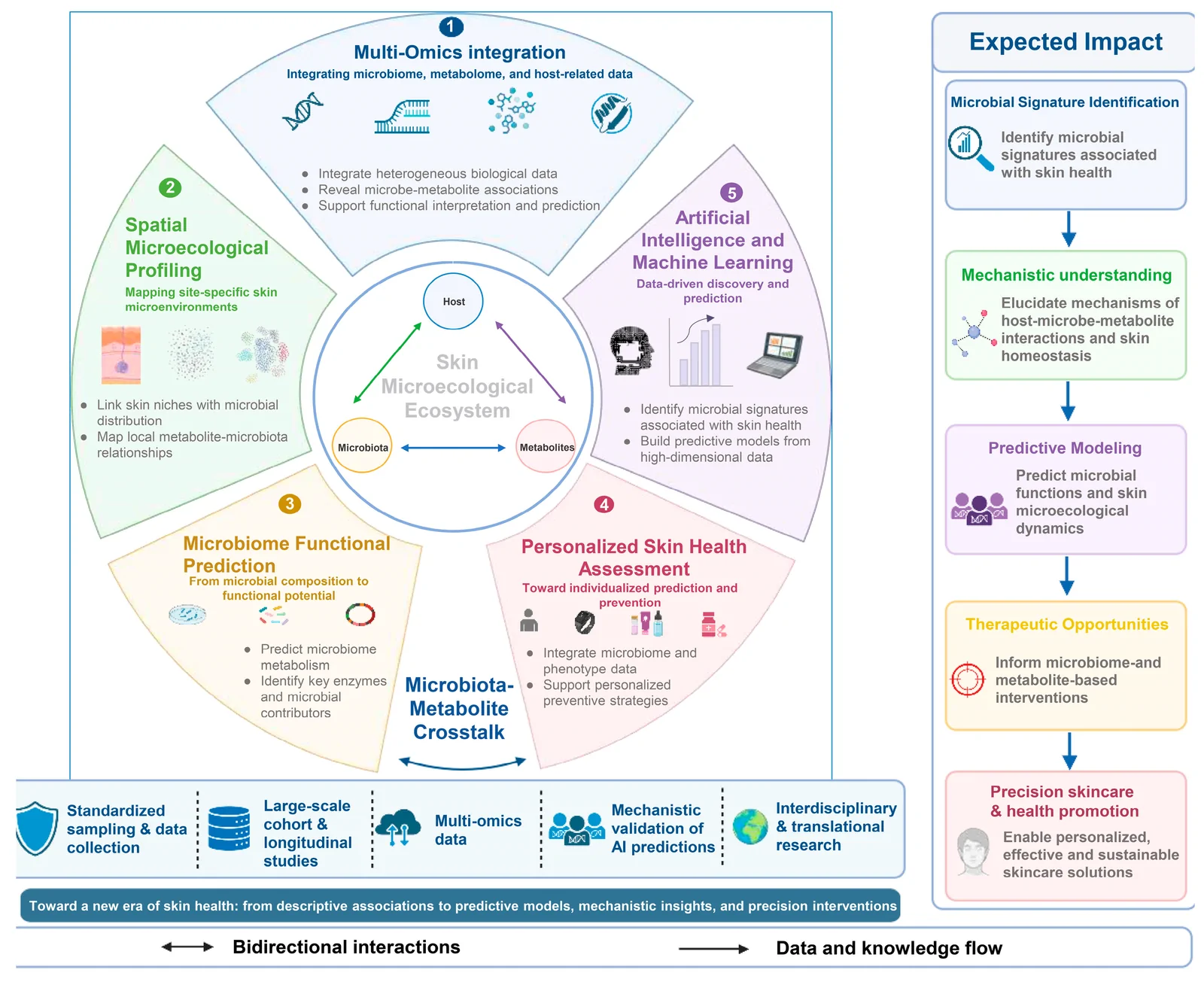
A multilevel framework for decoding skin microecology, from biological foundations to AI-enabled applications. The framework integrates spatial microecological profiling, multi-omics analysis, microbiome functional prediction, personalized skin health assessment, and artificial intelligence and machine learning around the central axis of metabolite–microbiota crosstalk. These interconnected approaches aim to advance skin microecology research from descriptive characterization toward mechanistic understanding, predictive modeling, and precision interventions. Standardized sampling and data collection, large-scale longitudinal studies, multi-omics data integration, mechanistic validation of AI predictions, and interdisciplinary collaboration provide essential support for this transition. Bidirectional interactions between metabolites and microbiota, together with the data and knowledge flow across analytical layers, constitute the driving force of this framework.

In this review, a multilevel perspective is adopted to synthesize current knowledge of the skin ecosystem. We first delineate how the skin’s unique architectural features create diverse microhabitats and spatially heterogeneous metabolic landscapes. We then examine the microbial communities that colonize these niches, with emphasis on their adaptive strategies to local metabolite milieus. We further systematically review the multifaceted roles of intrinsic metabolites, including lipids, proteins/peptides, ions, vitamins, and carbohydrates, as the chemical language mediating host–microbe crosstalk. Finally, we discuss how emerging AI-enabled approaches are poised to integrate these multi-level datasets, transforming our understanding from descriptive characterization to predictive modeling of skin microecological dynamics. This review synthesizes current knowledge across interconnected layers of the skin ecosystem, including architecture, microbiome, metabolome, and computational analytics, to provide a foundational framework for future investigations and to pave the way for the development of novel preventive and therapeutic strategies (Figure 1).

### Review Methodology and Scope

This narrative review aims to provide a comprehensive and structured overview of skin architecture, microbial communities, intrinsic metabolites, and emerging AI-enabled approaches to skin microecology. By integrating these interconnected areas, the review examines the relationships among skin structure, microbial communities, host- and microbiota-derived metabolites, and AI-assisted analytical approaches, while highlighting current knowledge gaps and future research directions.

The relevant literature was identified through non-systematic searches of PubMed and Web of Science, supplemented by searches in Google Scholar. Searches were conducted using combinations of terms including “skin architecture”, “skin barrier”, “skin microbiome”, “skin microecology”, “cutaneous microbiota”, “skin metabolites”, “microbial metabolites”, “host–microbe interactions”, “skin metabolomics”, “artificial intelligence”, “machine learning”, “deep learning”, and “multi-omics”. The primary focus was placed on peer-reviewed articles published between 2016 and 2026, while earlier foundational studies were included where necessary to provide historical or mechanistic context.

Studies were selected based on their relevance to the scope of this review, including research on human skin architecture and barrier function; the composition, spatial distribution, and functional characteristics of skin microbial communities; host- and microbiota-derived metabolites and host–microbe interactions; and AI, machine learning, or related computational approaches applied to microbial composition, microbial functions, skin phenotypes, metabolic profiles, or multi-omics data. When multiple studies addressed similar themes, preference was given to those with greater relevance, methodological rigor, representative study populations, and clearer mechanistic or functional evidence. For AI-related studies, additional consideration was given to model validation, interpretability, and the integration of microbiome, metabolome, or other multi-omics data.

As a narrative review, this work does not aim to exhaustively capture all published studies or provide a quantitative meta-analysis. Instead, it seeks to integrate representative and relevant evidence into a coherent multilevel framework that highlights the interconnected relationships among skin architecture, microbial communities, metabolites, and emerging AI-enabled approaches. To maintain coherence with our central focus on the metabolite–microbe–host axis, AI approaches were selected based on their direct or indirect relevance to microbial metabolism and skin microecology. Nevertheless, the non-systematic nature of this narrative approach may introduce a risk of selective citation and may not capture all relevant studies in this rapidly evolving field.

## 2. Skin Architecture and Its Role as a Metabolic Landscape for Microbial Habitats

From an anatomical perspective, the skin structurally consists of three distinct layers: the epidermis, dermis, and subcutaneous tissue. Beyond serving as a passive physical barrier, both the epidermis and dermis, together with their appendageal structures, function as metabolically active compartments that produce a diverse array of small molecules, ions, and macromolecules. These chemical outputs collectively establish a spatially heterogeneous microenvironment that profoundly shapes microbial colonization patterns.

### 2.1. The Epidermis: A Stratified Barrier with Metabolic Output

As the outermost layer, the epidermis is primarily composed of four strata: the stratum corneum (SC), stratum granulosum, stratum spinosum, and stratum basale. The SC, being the outermost stratum, consists of multiple layers of anucleate keratinocytes and varies in thickness from approximately 10–30 μm in most body regions, being considerably thicker on the palms and soles [13]. Beyond its well-recognized barrier function, the SC serves as the site where epidermal lipids and proteins, which are synthesized by differentiating keratinocytes in the granular layer and secreted via lamellar bodies, are processed and organized to create a nutrient-rich yet selective environment for microbial colonization. The lipid composition of the SC not only contributes to barrier integrity but also provides carbon sources for commensal microorganisms that possess lipolytic enzymes.

Beneath the SC, adjacent keratinocytes within the granular layer form tight junctions between themselves. Together with the SC, these tight junctions create an antibacterial barrier that prevents water loss while selectively allowing the passage of certain substances. Within the granular layer, keratinocytes undergo terminal differentiation, during which they synthesize keratohyalin granules containing profilaggrin, the precursor of filaggrin. Profilaggrin is subsequently processed into filaggrin, which is further degraded into natural moisturizing factors (NMFs), including urocanic acid, pyrrolidone carboxylic acid, and hygroscopic amino acids such as histidine and serine. NMFs help maintain hydration and pH balance, creating physicochemical conditions essential for sustaining a stable and diverse microbiota. In particular, the acidic environment generated by NMFs selectively inhibits pathogenic bacteria such as *Staphylococcus aureus* while favoring commensal species like *Staphylococcus epidermidis* [14,15]. In parallel, granular layer cells synthesize structural lipids that not only contribute to the formation of the SC but also serve as additional carbon sources and nutrients for commensal microorganisms. Collectively, the formation of this compact, lipid-rich, and mildly acidic interface establishes the physicochemical conditions that define the epidermis as a unique microbial habitat.

The stratum spinosum, composed of several layers of keratinocytes, provides structural support for this upper barrier. In this layer, cells form intercellular bridges that develop into desmosomes, which are strong adhesion structures that enhance the skin’s mechanical strength [16,17]. This robust structural framework helps limit microbial invasion and supports a stable physical environment for the surface microbiota. Beyond this structural role, keratinocytes in the spinous layer also participate in innate immune defense by producing antimicrobial peptides (AMPs) such as psoriasin (S100A7) and human β-defensins, which directly modulate microbial colonization by exerting selective pressure on the resident community [18,19].

The stratum basale, the innermost layer of the epidermis, consists of a single layer of proliferative basal keratinocytes that are anchored to the underlying basement membrane via hemidesmosomes. These cells are responsible for generating new keratinocytes through mitosis and synthesizing keratin. Melanocytes, which originate from the neural crest and reside in the stratum basale, produce melanin to provide pigmentation and UV protection. The basement membrane zone, together with the basal layer, forms the dermal-epidermal junction, which facilitates the exchange of nutrients and metabolic waste and plays a crucial role in maintaining microecological homeostasis [20]. However, the precise mechanisms by which basal layer-derived signals influence the overlying microbial communities remain incompletely understood and warrant further investigation.

### 2.2. The Dermis: Immune Surveillance and Structural Support

In contrast to the epidermis, which serves as the body’s primary physical and chemical barrier against microbial invasion, the dermis plays a more indirect yet equally important role in both structural support and immune surveillance. The dermis is 1 to 2 mm thick in most body regions and comprises two principal layers: the superficial papillary layer and the deeper reticular layer. Blood vessels, nerves, and lymphatic vessels pass through the dermis, facilitating nutrient exchange and immune cell trafficking. The dermis contains various immune cells, including dermal dendritic cells (DCs), macrophages, mast cells, γδ T cells, and innate lymphoid cells [21], which constantly survey the microbial landscape and respond to potential threats. Fibroblasts within the dermis produce collagen and elastin, providing the structural scaffold for leukocyte migration [19]. These immune cells are not only responsive to microbial signals but also actively shape the local metabolic milieu through the release of cytokines and antimicrobial effectors, thereby influencing the chemical environment that determines microbial survival and composition. This immune-competent compartment ensures that microbial breaches are rapidly detected and contained, thereby maintaining the overall stability of the skin ecosystem.

### 2.3. Appendageal Structures: Metabolically Active Niches

Within the dermis exists a range of physiologically and topographically distinct appendageal structures, including sweat glands, sebaceous glands, and hair follicles. Approximately 25% of the cutaneous bacterial population resides in these niches [22]. These specialized glands are not merely passive shelters; they function as metabolically active compartments that secrete sweat and sebum, which collectively form the acid mantle—a protective, slightly acidic film on the skin’s surface. This biochemical barrier provides essential nutrients for commensal microbiota and plays a crucial role in maintaining skin homeostasis by regulating moisture levels and creating an environment that discourages pathogenic microorganisms.

Sebaceous glands, which are abundant on the face, scalp, and upper trunk, secrete lipids including triglycerides, wax esters, squalene, and cholesterol [23]. These lipid-rich secretions serve as the primary carbon and energy sources for lipophilic microorganisms such as *Cutibacterium* and *Malassezia*. In turn, microbial lipases break down these lipids, releasing free fatty acids that contribute to the skin’s acidic pH and exert antimicrobial effects. Sweat glands, by contrast, produce an aqueous secretion containing lactate, urea, electrolytes (Na^+^, K^+^, Cl^−^), and antimicrobial peptides such as dermcidin [24,25]. Although sweat itself is near-neutral (pH 6.5–7.0), its lactate content contributes to the acid mantle, which is formed through the combined action of sweat, sebum-derived free fatty acids, and stratum corneum metabolites [26]. This distinct chemical composition creates a microenvironment that favors salt-tolerant and moisture-adapted taxa such as *Corynebacterium*. Hair follicles, particularly the deeper infundibulum and isthmus regions, create relatively anaerobic conditions due to limited oxygen diffusion, sebum accumulation, and oxygen consumption by resident microorganisms [27], providing niche environments for facultative anaerobes including *Cutibacterium acnes*. Thus, each appendageal structure generates a distinctive chemical signature that selectively favors microbial taxa equipped with the corresponding metabolic machinery. The spatial proximity of these distinct glands within the same topographical region creates a complex mosaic of metabolic microenvironments, collectively enriching the diversity of microbial habitats [11].

### 2.4. From Architecture to Metabolic Landscape

In summary, the skin’s architectural features, from the stratified epidermis to the immune-competent dermis and the metabolically active appendages, do not merely provide passive shelter for microorganisms. Rather, each structural compartment generates a distinct repertoire of metabolites that collectively establish a spatially heterogeneous chemical landscape. This integrated metabolic output, together with the skin’s physical topography and immune surveillance capacity, defines the fundamental conditions for microbial colonization and selection. Microbial communities have evolved diverse adaptive strategies to exploit these distinct metabolic niches, and their composition is largely dictated by the local chemical environment rather than by physical space alone.

## 3. Microbial Colonization of the Skin: Adaptive Strategies to Metabolic Niches

**Figure 2:**
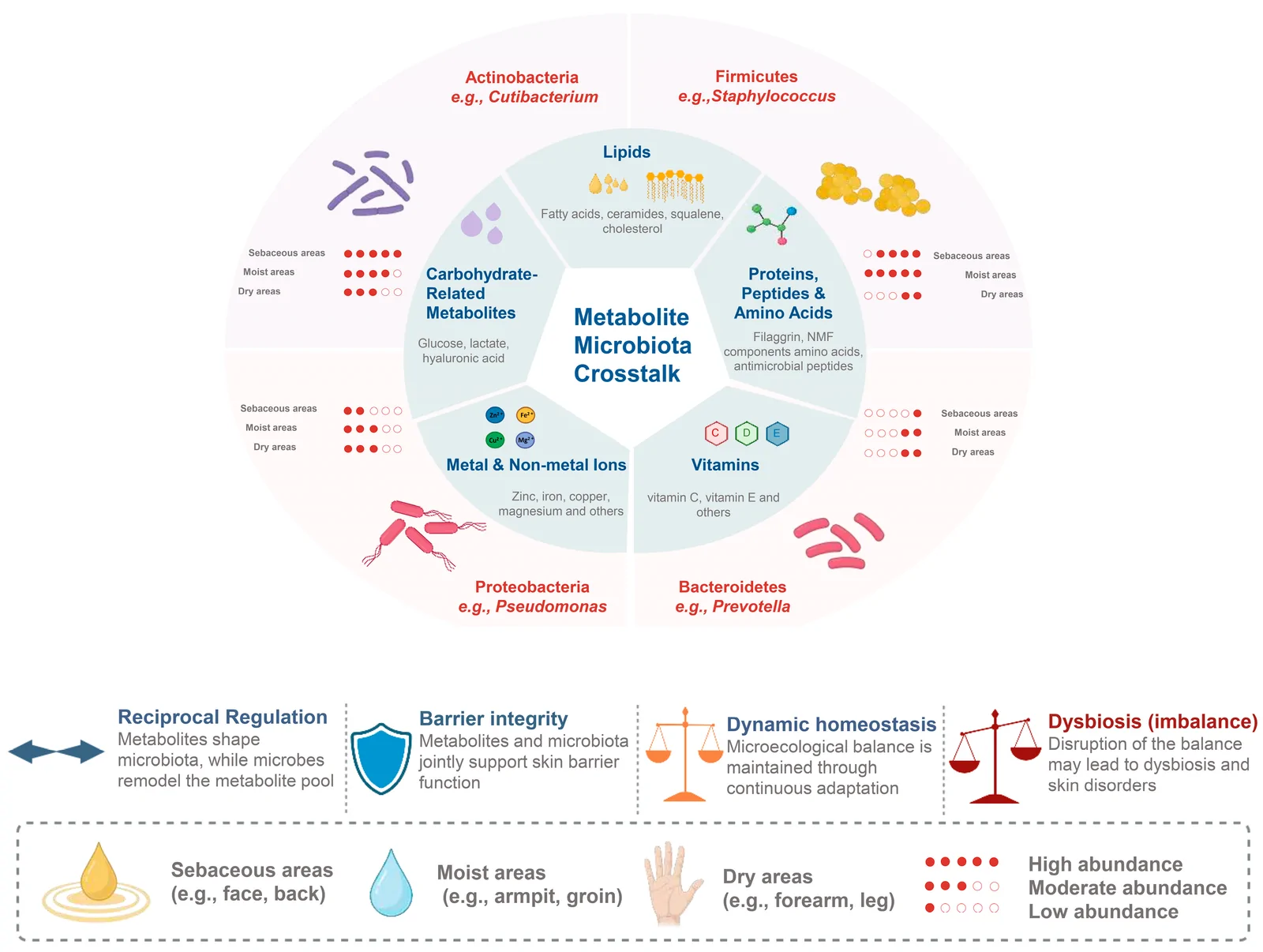
Bidirectional crosstalk between skin metabolites and microbiota across distinct skin microenvironments. Major classes of skin metabolites, including lipids, amino acids and peptides, proteins, vitamins, metal and non-metal ions, and carbohydrate-related metabolites, interact reciprocally with dominant microbial taxa. The composition and abundance of microbial communities vary across sebaceous, moist, and dry skin sites, with the relative abundance of major bacterial phyla differing markedly among these regions. Site-specific microenvironmental conditions, particularly the availability of lipids, moisture, and antimicrobial factors, shape these distribution patterns.

The skin surface of adults, measuring approximately 1.8 m^2^, is colonized by nearly 1 million bacteria per cm^2^ [21]. The skin harbors complex microecosystems of bacteria, fungi, viruses, and mites, each with distinct adaptations to survive on the skin. Among these microorganisms, bacteria are the most abundant and have been the most extensively characterized. Four major bacterial phyla colonizing the skin have been identified: Actinobacteria (51.8%), Firmicutes (24.4%), Proteobacteria (16.5%), and Bacteroidetes (6.3%) [11]. Notably, these phyla are not uniformly distributed; their relative abundance varies considerably across sebaceous, moist, and dry body regions, reflecting differential adaptations to locally available metabolite resources (Figure 2).

### 3.1. Bacterial Communities: Metabolic Adaptations to Diverse Microenvironments

Sebaceous areas, which are rich in fatty acids and amino acids, are predominantly inhabited by *Cutibacterium* and *Staphylococcus*. *Cutibacterium*, a facultative anaerobe, utilizes proteases to release amino acids from skin proteins and lipases to degrade the triglycerides secreted in sebum. This lipolytic activity is not merely a nutritional strategy; it actively transforms the local lipid environment. Conversely, *Cutibacterium*-derived short-chain fatty acids can stimulate epidermal lipid synthesis, increasing triglycerides, ceramides, cholesterol, and free fatty acids, thereby reshaping the cutaneous lipid microenvironment. *Staphylococcus* species, such as *Staphylococcus aureus* and *Staphylococcus epidermidis*, tolerate the low pH of sebaceous sites and produce lipases to utilize lipid-rich substrates [19]. The ecological outcomes of these two staphylococcal species, however, diverge considerably. While *Staphylococcus aureus* can act as an opportunistic pathogen, *Staphylococcus epidermidis* plays a beneficial role by secreting sphingomyelinase, which increases skin ceramide levels and prevents water loss in damaged skin [28]. The retroauricular crease, another sebaceous site, exhibits relatively stable composition due to the prevalence of *Cutibacterium*.

Moist areas, such as the antecubital fossa, inguinal crease, and popliteal fossa, are characterized by high temperature and humidity and serve as main habitats for *Corynebacterium* and *Staphylococcus* [29]. *Corynebacterium*, a Gram-positive bacterium, thrives in high-salt environments, typically absorbs lipids from the skin, and utilizes sweat as a source of vitamins. Corynebacterium utilizes these lipids as carbon and energy sources and synthesizes corynemycolic acids that are incorporated into the cell wall, contributing to an outer permeability barrier that restricts the penetration of hydrophilic antibacterial agents. This osmotolerant and lipid-metabolizing capacity, combined with its ability to synthesize corynemycolic acids, enables *Corynebacterium* to dominate in sweat-enriched, moisture-rich niches where other taxa are excluded by osmotic stress.

Dry areas, such as the hypothenar palm and volar forearm, exhibit high microbial diversity but low temporal stability, and are predominantly inhabited by *Cutibacterium* [11]. The relatively sparse nutrient availability and variable hydration levels in these regions may favor metabolically versatile taxa capable of switching between different substrate sources, although the ecological factors underlying the high diversity but low stability of dry sites remain incompletely understood.

### 3.2. Fungal Communities: Lipophilicity-Driven Dominance

Fungal diversity on human skin is generally lower than bacterial diversity. *Malassezia* spp. are the most important fungi colonizing human skin and are primarily distributed in sebum-rich areas, such as the scalp, face, chest, and back, accounting for approximately 50–80% of the total fungi population in healthy individuals [1,30]. Metagenomic analysis has revealed that the *Malassezia* genome is enriched in lipase genes but lacks carbohydrate-utilizing enzyme genes, which explains why *Malassezia* species predominate in sebum-rich areas and illustrates how metabolite composition filters microbial membership at the genetic level. Several skin disorders are associated with *Malassezia*, the most common being pityriasis versicolor and seborrheic dermatitis. Additionally, an imbalance in *Malassezia* proportions might contribute to androgenetic alopecia (AGA), the most common hair loss disorder. Beyond *Malassezia* spp., emerging pathogens such as *Candida albicans* and *Candida auris* have been found to persist on the skin surface as well as in deeper tissue layers [31], though their ecological roles in healthy skin remain less well characterized.

### 3.3. Mites and Viruses: Additional Layers of Microbial Complexity

Approximately 65 species of *Demodex* have been described, with two species—*Demodex folliculorum* and *Demodex brevis*—found in human skin [9,32]. Both possess three primary body segments (gnathosoma, podosoma, and opisthosoma) and feed on secretions from sebaceous glands. *Demodex folliculorum*, ranging from 0.3 to 0.4 mm in length, primarily resides in the hair follicles of various facial areas, such as the cheeks, forehead, nose, and eyes, as well as the eyebrows and eyelashes. *Demodex brevis*, which is smaller at 0.15–0.2 mm in length, inhabits sebaceous glands on the face, neck, and torso, particularly in the meibomian glands of the eyelids [33]. The presence of *Demodex* is associated with numerous ocular and skin disorders, including perioral dermatitis, pustular folliculitis, and rosacea or rosacea-like dermatitis. The incidence of *Demodex* infestation rises with age. Notably, the metabolic interactions between *Demodex* and the surrounding microbial community, particularly with *Cutibacterium* and *Malassezia* within the same sebaceous niches, remain poorly understood and warrant further investigation.

Viruses also play a significant role in maintaining the homeostasis of the skin microbiota. Due to the low biomass present in skin samples, research on viruses requires advanced techniques [34]. The types of viruses on sebaceous, moist and dry areas of the skin differ significantly. The identified top ten viruses across the three skin sites include *Acheta domestica Densovirus*, *Alphapapillomavirus*, *Human papillomavirus* (β, γ and μ), *Merkel cell polyomavirus*, *Molluscum contagiosum virus*, *Polyomavirus HPyV7*, *Polyomavirus HPyV6*, *RD114 retrovirus* and *Simian virus* [9,35].

Among the characterized eukaryotic viruses, *Molluscum contagiosum virus* (MCV) is frequently detected in the moist and dry areas of human skin. However, it is important to note that the healthy skin virome is highly diverse and is predominantly composed of bacteriophages, with eukaryotic viruses representing a smaller fraction. MCV is a double-stranded DNA virus with a brick-shaped morphology. Humans are the only hosts for MCV, and it can cause molluscum contagiosum in human skin, a highly contagious and infectious dermatologic disease commonly seen in children, sexually active young adults, and immunosuppressed patients, with a worldwide incidence of approximately 8000 cases per 100,000 individuals [36]. Individuals with skin barrier dysfunction, such as in atopic dermatitis, are more susceptible to MC [37]. The interactions between viral infection and the skin metabolic environment, however, remain largely unexplored, representing a substantial gap in our understanding of how viruses integrate into the skin’s chemical ecology.

### 3.4. Metabolic Niche Partitioning as a Determinant of Microbial Biogeography

The distinct distribution patterns of bacterial, fungal, viral, and mite communities across skin sites can be understood as differential adaptations to locally available metabolite resources. Lipophilic genera exploit lipid-rich sebaceous milieus, osmotolerant taxa thrive in moist, sweat-enriched environments, and metabolically versatile taxa dominate nutrient-scarce dry regions. This metabolic niche partitioning underscores a central principle: the skin’s chemical environment, rather than physical space alone, is a primary determinant of microbial biogeography. Importantly, the host–microbe relationship is bidirectional; microbial metabolism, through enzymatic activities such as lipolysis, proteolysis, and sphingomyelin hydrolysis, reciprocally remodels the local metabolite pool, thereby influencing the chemical landscape that in turn shapes community succession. Thus, the spatial and temporal dynamics of the skin microbiome cannot be fully understood without considering the metabolic context in which microbial communities are embedded.

## 4. Skin Metabolome: The Chemical Language of Host–Microbe Dialogue

For decades research has demonstrated that no single tool can fully elucidate the composition of an ideal balanced skin microbiome. This poses a significant challenge for developing highly targeted therapeutics to prevent skin disease or promote microecological balance, as the diversity and stability of the skin microbiome are dynamically influenced by microenvironmental factors and host-microorganism interactions. Nevertheless, relatively stable core skin microbial taxa do exist, yet community composition varies among individuals and is jointly determined by local skin physiology, genetics, microbe-host interactions, and microbe-microbe interactions. Among these determinants, skin metabolites are the most direct factors affecting community composition and maintaining skin microbiome balance. Intrinsic skin metabolites are not merely passive nutrients; they constitute the chemical language through which the host signals to its microbial partners and through which microbes reciprocally influence host physiology.

**Figure 3:**
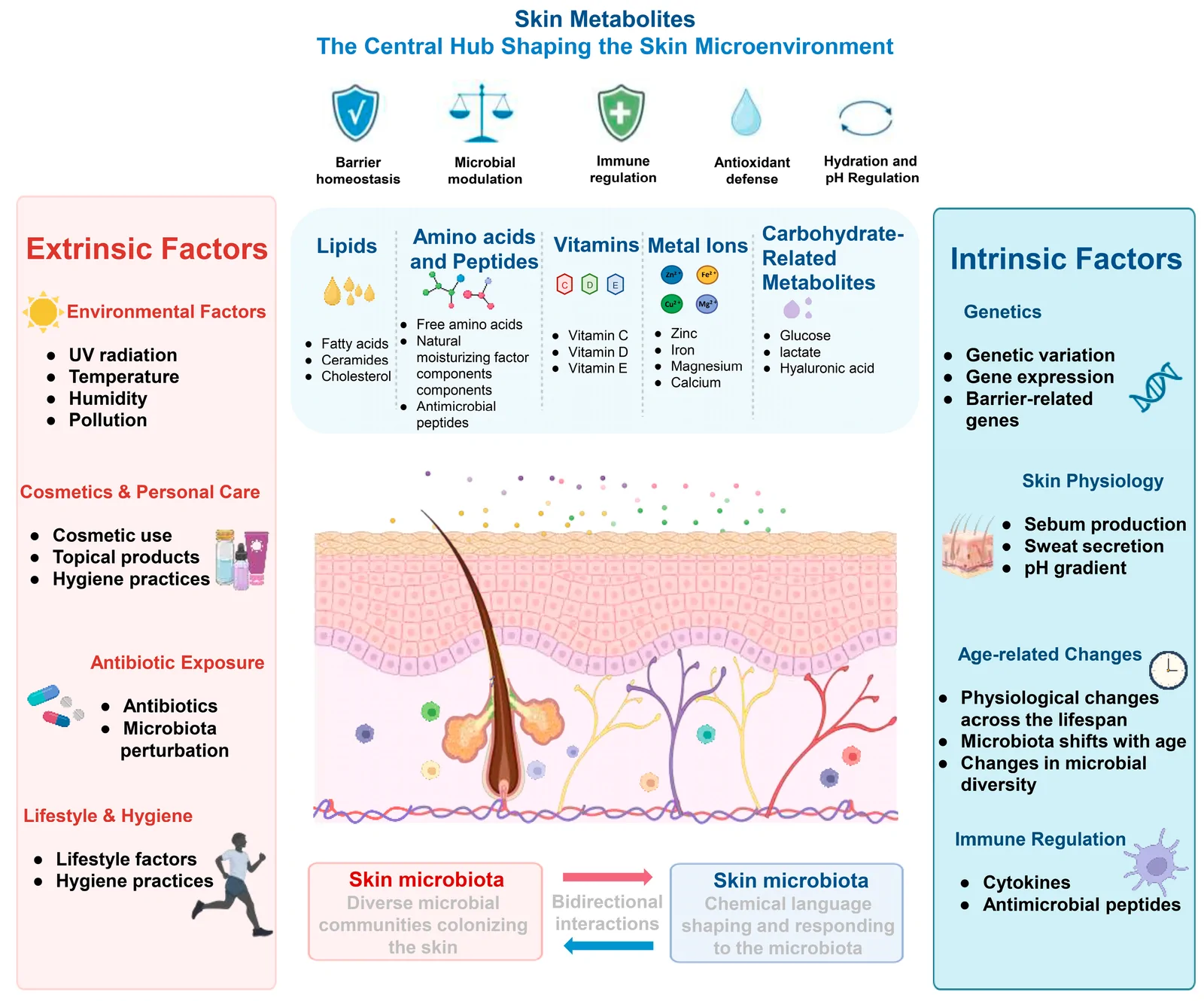
Intrinsic and extrinsic factors shaping skin metabolite–microbiota interactions and the skin microenvironment. Major classes of skin metabolites, including lipids, amino acids and peptides, vitamins, metal ions, and carbohydrate-related metabolites, interact bidirectionally with the resident microbiota and collectively constitute the chemical language of host–microbe communication. These metabolites contribute to barrier homeostasis, microbial modulation, immune regulation, antioxidant defense, hydration, and pH regulation. These interactions are dynamically shaped by intrinsic factors, including genetics, skin physiology, age-related changes, and immune regulation, as well as extrinsic influences such as environmental factors, cosmetics and personal care practices, antibiotic exposure, and lifestyle and hygiene. The skin microbiota, in turn, both responds to and reshapes this chemical environment, establishing a reciprocal feedback loop that sustains microecological balance.

Human skin metabolites encompass a diverse array of small molecules derived from multiple sources, including epidermal keratinocytes, sebaceous and sweat glands, and microbial metabolism. These intrinsic metabolites can be broadly categorized into several major chemical classes: lipids and their metabolites, metal and non-metal ions, amino acids and their derivatives, peptides, vitamins, and carbohydrates. Their composition and spatial distribution are further modulated by extrinsic factors such as mode of delivery, lifestyle, hygiene practices, cosmetic use, antibiotic exposure, geographical location, climate, and seasonality (Figure 3).

### 4.1. Lipids

Lipids and their metabolites are major components of the human skin metabolome. Based on their sources, they can be classified into five types: sweat-derived lipids, epidermal lipids (from the SC), sebaceous lipids (from the sebaceous glands), plasma lipids, and lipids originating from cosmetics or personal care products [12]. Based on their functions, they can be categorized into three groups: physicochemical functions, biochemical functions, and microecological functions.

This regulatory effect of skin lipids on microecological balance is bidirectional. On one hand, skin lipids serve as a nutritional source for the microbiome, providing free fatty acids (FFAs), glycerol, and cholesterol as carbon sources for skin microorganisms, although the degree of dependence on specific lipid types varies among different microbial species [38,39]. For example, *Corynebacterium jeikeium* depends almost completely on host lipids for survival, whereas *Staphylococcus aureus* employs the fatty acid kinase system (FakAB) to activate exogenous fatty acids. On the other hand, in addition to their nutritional role, skin lipids also possess antimicrobial properties. For example, sphingosine could bind to bacterial membrane cardiolipin, thereby disrupting cell integrity [40].

Several members of the microbiome can secrete lipase, sphingomyelinase, and ceramidase to degrade phospholipids, sphingolipids, and triacylglycerols. For instance, *Staphylococcus aureus* utilizes the enzyme GlpQ to release glycerol for energy metabolism [41]. Notably, different microbes exhibit varying sensitivities to lipids, which determines their competitive capacity within the skin microecosystem. For example, streptococci are more vulnerable than *S. aureus* to antimicrobial free fatty acids (AFAs) and monoacylglycerols [42]. *Staphylococcus epidermidis* can utilize sphingomyelinase to increase ceramide content in the skin, helping to maintain barrier function. In contrast, *Cutibacterium acnes*-derived short-chain fatty acids can stimulate epidermal lipid synthesis, increasing triglycerides, ceramides, cholesterol, and free fatty acids, thereby reshaping the cutaneous lipid microenvironment and potentially contributing to acne-associated changes [43]. Specific microbe–metabolite pairs illustrate this bidirectional regulation. For example, S. epidermidis secretes sphingomyelinase, which hydrolyzes host sphingomyelin to generate ceramides, thereby directly strengthening the skin barrier. Conversely, *C. acnes* produces short-chain fatty acids that stimulate epidermal lipid synthesis, which in turn alters the lipid substrates available to the microbial community. These examples demonstrate how individual microbial metabolic activities can reshape the lipid landscape and, consequently, the ecological conditions for other community members. Collectively, these examples illustrate that skin lipids are not merely passive nutrients but active participants in a bidirectional regulatory network that links microbial metabolism to host barrier function.

### 4.2. Peptides, Proteins, and Their Derivatives

Similar to lipids, proteins, peptides, and their derivatives play a vital role in human skin, influencing the balance of the skin microecology. Protein degradation is a noteworthy process that contributes to the renewal of the skin’s layered structure throughout the lifespan, with filaggrin being one of the most significant factors involved [44]. Filaggrin is a protein isolated from the SC, first described in 1981 by Steinert and colleagues, and is a member of the S100 fused-type protein family [45,46]. Filaggrin is involved in a variety of functions, including contributing to SC integrity, serving as a source of NMFs, and regulating the microecological balance of human skin [47]. Following its synthesis as profilaggrin and proteolytic processing into filaggrin monomers, filaggrin is cleaved by multiple proteases, including caspase-14, into free amino acids and their derivatives. Subsequent enzymatic metabolism of these amino acids yields hygroscopic metabolites, including urocanic acid (from histidine) and pyrrolidone carboxylic acid. These metabolites, together with lactate, hyaluronic acid, and inorganic ions (Na^+^, K^+^, Ca^2+^, and Mg^2+^), constitute the natural moisturizing factor (NMF) of the skin [47]. The degradation of filaggrin into NMF components represents a critical node in the host–microbe interface, as the resulting metabolites directly modulate the physicochemical conditions that determine which microorganisms can colonize the skin surface.

Loricrin is another major structural protein that contributes to the mechanical resilience of the SC. While loricrin itself primarily serves a structural role, its degradation products also contribute to the pool of free amino acids on the skin surface, thereby indirectly influencing microbial nutrition. Loricrin deficiency has been shown to increase DNA damage and oxidative stress, which in turn alters the skin microecology and affects microbial colonization [48]. Keratins are major intermediate filament proteins that provide mechanical support and structural integrity to epidermal keratinocytes [49].

In addition to the proteins derived from the SC, neuropeptides from sweat glands are also important. These include substance P(SP), a member of the tachykinin neuropeptide family that mediates neuroimmune and inflammatory responses, and calcitonin gene-related peptide (CGRP), which can sense microbes, is critical for skin homeostasis, and modulates skin microbiome virulence [50]. In contrast to neuropeptides, antimicrobial peptides (AMPs) derived from sweat, sebocytes, and keratinocytes (KCs) participate in epithelial innate defense, directly defending against skin microorganisms. For example, one of the main biological functions of epithelial S100 proteins, a type of AMP, is to selectively target microbial growth and kill potentially pathogenic microbes on body surfaces by sequestering essential transition metal ions [51].

Beyond their structural roles, the degradation products of these proteins exert profound effects on the skin microecology. Notably, serine—the most abundant constituent of NMF, accounting for more than one-fifth of the total free amino acids in NMF—plays an important role in skin hydration owing to its remarkable water-retention capacity. In addition to this function, serine also contributes to immune responses by regulating skin inflammation and promotes keratinocyte turnover, thereby supporting the renewal of the SC. Thus, epidermal serine content can serve as a valuable indicator of skin health, and disturbances in serine metabolism may trigger various skin disorders, including psoriasis [52,53].

Besides serine, histidine is also a component of NMF, and its level is closely associated with skin pH. Higher NMF levels in the skin create an acidic environment that inhibits pathogenic microbiota such as *Staphylococcus aureus* while favoring the stable colonization of commensal species such as *Staphylococcus epidermidis* [24]. NMF is crucial for SC hydration and acidity, thereby supporting skin barrier function. Consistently, histidine supplementation has been shown to significantly improve the symptoms of atopic dermatitis (AD) in both children and adults, as AD patients often have filaggrin deficiency, and histidine is a key constituent of filaggrin. In addition to its moisturizing role, histidine also contributes to the skin’s UV defense system. Under the action of histidase, the first enzyme in its metabolic pathway, histidine is converted into trans-urocanic acid (trans-UCA). Trans-UCA acts as a natural UV-absorbing agent, protecting DNA from UVB-induced damage (270–320 nm). Upon UV exposure, trans-UCA can be nonenzymatically photoisomerized into cis-urocanic acid (cis-UCA). Unlike trans-UCA, cis-UCA does not primarily function as a UV filter; instead, it exerts immunosuppressive effects and is implicated in UV-induced immune suppression [54,55]. Thus, the dynamic balance between trans- and cis-UCA may serve as a key photobiological link between UV exposure, cutaneous immune regulation, and microecological stability.

A clear example of microbe–metabolite crosstalk is the interplay between filaggrin-derived histidine and *S. aureus*. The acidic environment generated by histidine-derived urocanic acid selectively inhibits *S. aureus* growth while favoring *S. epidermidis*. Thus, proteins and their derivatives serve dual functions: as structural components that maintain barrier integrity and as a reservoir of bioactive small molecules that directly modulate microbial community composition through pH regulation, nutrient provision, and antimicrobial activity.

### 4.3. Metal and Non-Metal Ions

Metal and non-metal ions are important components of the endogenous metabolic milieu of the skin and play indispensable roles in skin microecology. They are involved not only in the maintenance of skin barrier homeostasis, wound repair, and immune regulation, but also in shaping the physicochemical environment of the skin and influencing microbial metabolic activity. Functionally, these ions contribute to the protection and renewal of the skin surface, the maintenance of structural integrity and elasticity, and the regulation of hydration, osmotic balance, and buffering capacity.

Sodium is stored in the skin interstitium and muscle in both free and non-osmotic forms, and its major function is to prevent water loss from the skin. Abnormalities in cutaneous sodium content have been observed in patients with acute cellulitis, supporting a role for sodium in skin infection. Potassium and sodium also contribute to the maintenance of hydration and osmotic balance in the skin. In addition, sulfur is involved in skin hydration and extracellular matrix (ECM) maintenance, thereby influencing skin texture and barrier properties.

Among the ions involved in skin injury repair, calcium, zinc, and magnesium are particularly important. Wound healing consists of four overlapping stages: hemostasis, inflammation, proliferation, and tissue remodeling [56]. In this process, calcium ions play diverse and critical roles by accelerating coagulation, modulating neutrophil function during inflammation, regulating keratinocyte proliferation and differentiation through calcium gradients and CaSR signaling, and promoting fibroblast activity and angiogenesis, thereby facilitating effective tissue repair [57]. Zinc promotes keratinocyte differentiation and regulates immune responses, both of which are essential for wound healing and epidermal barrier maintenance. Notably, the skin is the third most zinc-abundant tissue in the human body, and sufficient zinc is required to maintain barrier function, hydration, and structural integrity [58]. Magnesium also contributes to skin hydration, tissue repair, and inflammatory regulation [59]. In addition to these ions, copper serves as an important factor in collagen metabolism and promotes elastin synthesis and angiogenesis, whereas selenium, as a cofactor of glutathione peroxidase (GPx), helps fibroblasts resist oxidative stress. In contrast, iron primarily contributes to oxygen transport to the dermis [60].

Beyond their roles in host physiology, metal ions directly influence microbial physiology and community structure. Iron availability, in particular, constitutes a central battleground in host–microbe competition: the host sequesters iron through transferrin and lactoferrin to limit bacterial growth, while *S. aureus* counteracts this nutritional immunity by secreting siderophores such as staphyloferrin. Zinc exerts direct antimicrobial activity at high concentrations and modulates bacterial biofilm formation, whereas copper and silver ions are well-established bactericidal agents that disrupt membrane integrity and enzyme function. Calcium ions influence bacterial adhesion and biofilm development, and magnesium serves as an essential cofactor for numerous microbial enzymes. These observations indicate that the skin’s ionic milieu is not merely a host-derived physiological parameter but an active determinant of microbial community composition.

From a microecological perspective, these ions exert selective pressures on microbial communities. For example, the high salt concentration in sweat creates an osmotically challenging environment that favors salt-tolerant taxa such as *Corynebacterium*, while calcium and zinc ions can directly influence bacterial adhesion and biofilm formation. Iron competition provides a particularly well-characterized example of host–microbe interaction via metal ions. The host sequesters iron through transferrin and lactoferrin, limiting its availability to pathogens; in response, *S. aureus* secretes siderophores such as staphyloferrin to acquire iron. This nutritional immunity arms race directly influences staphylococcal colonization and virulence [61]. Together, these metal and non-metal ions form an essential part of the skin’s endogenous chemical microenvironment and collectively support skin microecological homeostasis.

### 4.4. Vitamins

Beyond the structural and regulatory roles of lipids, proteins, amino acids, metal ions, and non-metal ions, vitamins provide unique regulatory capacities, particularly in antioxidant defense, barrier maintenance, and host–microbe interactions within the skin ecosystem.

Normal skin requires high concentrations of vitamin C, which indicates active accumulation. Higher levels of vitamin C are found in the epidermis than in the dermis, with the epidermal content being approximately 425% greater than that of the dermis [62]. Vitamin C exerts multiple functions in the skin, such as resisting UV-induced oxidative stress, inhibiting melanogenesis, and promoting keratinocyte differentiation, which highlights its important role in skin microecology [63]. The human skin possesses a complex antioxidant defense system, which is divided into two categories: the enzymatic antioxidant system and the non-enzymatic antioxidant system, including vitamin C, vitamin E, and glutathione. Vitamin C can inhibit the increase in matrix metalloproteinase-1 (MMP-1) to prevent collagen degradation, as MMP-1 is the major collagenolytic enzyme responsible for collagen damage in UV-irradiated skin, especially under UVA exposure [64]. The combined use of vitamin C and vitamin E significantly decreases oxidative damage in the skin, while both also contribute to the inhibition of melanogenesis [65,66,67,68].

In contrast to vitamin C, vitamin E must be obtained from dietary sources, as it is synthesized by plants. Vitamin E can be divided into eight categories, while the content of γ-tocopherol exceeds that of α-tocopherol in human skin, which can prevent sunburn cell formation, ultraviolet (UV) B-induced lipid peroxidation and edema [69]. In addition to this function, vitamin E plays an important anti-inflammatory role. Vitamin E not only neutralizes free radicals but also modulates signaling pathways, cytokines, kinases, and enzymes involved in inflammation and oxidative stress [70].

Emerging evidence suggests that vitamins also participate in host–microbe communication. Vitamin D receptor signaling in keratinocytes modulates the expression of antimicrobial peptides such as cathelicidin and β-defensins, thereby indirectly shaping the skin microbial community. Vitamin B3 (niacinamide) exerts anti-inflammatory effects that may influence the cutaneous environment available for microbial colonization. Vitamin A and its derivatives regulate sebocyte differentiation and sebum production, thereby altering the lipid substrates available to lipophilic microorganisms such as *Cutibacterium* and *Malassezia*. These observations suggest that vitamins are not merely host-directed antioxidants but also indirect modulators of the skin’s microbial ecology.

Beyond its antioxidant and anti-inflammatory roles, vitamin E complements vitamin D, which contributes to the maintenance of skin barrier integrity, thereby highlighting their synergistic functions in sustaining skin microecology. The interaction between calcitriol and the vitamin D receptor (VDR) plays a significant role in the skin’s immune system, as it promotes the synthesis of ceramides, which are vital components required for preserving the integrity and functionality of the epidermal barrier [71,72]. In addition, calcitriol can enhance intracellular calcium concentrations, thereby promoting the activation of structural proteins and ultimately facilitating keratinocyte differentiation [73,74]. One of the best-characterized examples is vitamin D-VDR signaling, which induces the expression of cathelicidin and β-defensins in keratinocytes. These antimicrobial peptides directly kill *S. aureus* and other pathogens, thereby linking a host vitamin metabolite to microbial community composition [75]. The roles of vitamins in modulating skin microbial communities, however, remain less explored than their canonical antioxidant and barrier functions and warrant further investigation.

### 4.5. Carbohydrate-Related Metabolites

Glucose, lactic acid, and hyaluronic acid are carbohydrates and carbohydrate-related metabolites that can influence the skin microecology.

The skin is an important source of lactic acid. Keratinocytes, sebocytes, and hair follicle stem cells all exhibit substantial lactate-producing capacity. In addition, members of the skin microbiota, such as *Cutibacterium acnes*, *Staphylococcus epidermidis*, and *Lactobacillus*, can utilize host-derived metabolites to promote lactic acid production. Lactic acid exists as two stereoisomers, L-lactic acid and D-lactic acid, and the ratio between them may reflect certain physiological or pathological conditions of the skin [76]. The stratum corneum exhibits a distinct pH gradient, characterized by a highly acidic lower layer, an acidic middle layer, and a near-neutral upper layer, which is critical for maintaining stratum corneum barrier function. As a weak organic acid, lactic acid exerts at least two important functions in the skin. First, it participates in pH regulation by acidifying the local cellular microenvironment. Second, it modulates keratinocyte apoptosis and influences keratinocyte differentiation. Under pathological conditions, lactic acid is considered an important factor in restoring skin barrier integrity. It also promotes wound healing by serving as an energy substrate and by lowering the local pH, thereby creating an acidic microenvironment that supports cell proliferation and differentiation within an optimal physiological pH range [77].

Elevated glucose levels provide skin microorganisms with additional carbon sources, thereby promoting microbial proliferation and increasing bacterial load. Under hyperglycemic conditions, the expression of Aureolysin, a virulence factor of *Staphylococcus aureus*, has been reported to increase by approximately twofold. The increase in glucose availability, together with the upregulation of Aureolysin, enhances bacterial virulence and promotes the degradation of collagen and fibronectin, thereby impairing skin barrier integrity [78].

Hyaluronic acid (HA) is a non-branched glycosaminoglycan capable of binding large amounts of water and is an important component of the skin. It exerts three major functions: hydration, viscoelasticity, and tissue homeostasis. A reduction in hyaluronic acid content may decrease skin hydration, thereby contributing to loosening of the stratum corneum and disruption of barrier integrity. In addition, hyaluronic acid regulates skin hydration, maintains stratum corneum barrier structure, and facilitates the retention of active substances. By shaping the physicochemical microenvironment of the skin surface, hyaluronic acid may further influence microbial colonization [79,80]. However, the direct effects of hyaluronic acid on specific microbial taxa remain to be elucidated. Lactic acid provides another example of microbe–metabolite crosstalk. *C. acnes* and *S. epidermidis* can utilize host-derived metabolites to produce lactic acid, which acidifies the local microenvironment and may selectively favor acid-tolerant taxa.

### 4.6. The Integrated Metabolic Network

The five classes of intrinsic metabolites, namely lipids, proteins/peptides, metal/non-metal ions, vitamins, and carbohydrates, do not function in isolation. Rather, they form an integrated chemical network that simultaneously provides nutritional support, transmits signaling cues, and enforces antimicrobial defense. Lipids supply carbon and energy while also exerting direct antimicrobial activity; protein-derived NMFs acidify the skin surface and provide essential amino acids; ions regulate osmotic balance and exert selective pressures on microbial communities; vitamins contribute antioxidant and barrier-maintenance functions; and carbohydrate-related metabolites modulate pH, hydration, and substrate availability. This network constitutes the metabolic foundation of skin microecological homeostasis. Perturbations in any of these metabolite classes, whether through genetic predisposition, environmental exposure, or microbial dysbiosis, can disrupt the finely tuned chemical equilibrium, leading to skin barrier dysfunction and disease. These metabolite classes are not merely additive; they interact synergistically to shape the microbial landscape. For example, the acidic pH generated by NMF-derived urocanic acid and lactic acid enhances the antimicrobial activity of free fatty acids, while calcium ions regulate the lipid synthesis pathways that produce these fatty acids. Similarly, histidine-derived urocanic acid and vitamin D-induced antimicrobial peptides act in concert to suppress *S. aureus* colonization. These synergistic interactions, rather than any single metabolite class in isolation, constitute the chemical language that governs skin microecological homeostasis. While the examples above illustrate specific microbe–metabolite–physiology links, it is important to recognize that much of the current evidence remains correlative. The presence of a microbial taxon and a metabolite in the same skin niche does not establish that the microbe produces or consumes that metabolite. Establishing causal links requires approaches such as stable isotope probing, targeted gene knockout, and defined co-culture experiments. For example, while *S. epidermidis* sphingomyelinase activity has been directly linked to ceramide production [28], the metabolic contributions of many other skin taxa remain inferred rather than experimentally demonstrated. This gap between correlation and causation represents a critical challenge for the field. The complexity and high-dimensional nature of this metabolic network, however, pose substantial challenges for traditional analytical approaches.

## 5. Emerging AI-Enabled Approaches for Decoding Skin Microecology

The skin microecosystem is characterized by multi-layered complexity, including structural heterogeneity, microbial diversity, and metabolic dynamics, all interacting in a highly non-linear and personalized manner. Traditional analytical approaches, while valuable for univariate or bivariate correlations, are fundamentally limited in capturing the high-dimensional, interactive networks that govern this ecosystem. This is where artificial intelligence (AI) and machine learning become indispensable. By integrating heterogeneous biological data, uncovering hidden patterns, and predicting functional crosstalk, AI-driven technologies open up new avenues for deciphering skin microbe-host interactions and advancing personalized skin health strategies.

It is important to clarify the relationship between AI applications and the metabolite-centered focus of this review. Current AI approaches in skin microecology operate at three levels of connection to metabolites. At the most direct level, tools such as SkinBug predict microbial metabolic functions—that is, which metabolites microbes can produce or transform. At an indirect level, machine learning models identify associations between microbial composition and host phenotypes (e.g., skin hydration, pH), with metabolites serving as inferred mediators that have not yet been directly measured. At a methodological level, techniques such as federated learning, transfer learning, and explainable AI provide the infrastructure needed to improve model reliability and interpretability, but do not themselves generate metabolite data. The relative scarcity of AI models trained directly on skin metabolomics data reflects both the technical challenges of metabolome profiling (e.g., low biomass, high chemical diversity) and the limited availability of paired microbiome–metabolome datasets. This evidence hierarchy should be kept in mind when interpreting the applications discussed below (Table 1).

### 5.1. AI-Driven Prediction of Microbiome-Mediated Metabolic Functions

Jaiswal et al. developed an AI-based prediction tool called SkinBug to elucidate biotransformation processes mediated by the skin microbiota. By integrating machine learning, artificial neural networks, and chemoinformatics approaches, this tool established a database containing 897 skin microbial pan-genomes and information on more than 1,094,000 metabolic enzymes, enabling the prediction of potential metabolic pathways of specific compounds within the skin microecological environment. Unlike traditional approaches that mainly focus on which microorganisms are present, SkinBug can further predict key enzymes involved in metabolic reactions, the microbial species carrying these enzymes, and their corresponding skin colonization sites, thereby facilitating the transition from microbial composition analysis to functional potential assessment. This study demonstrates that the skin microbiome is not merely a colonizer of the skin surface but also possesses complex metabolic capabilities, enabling the transformation of host-derived molecules and exogenous compounds (such as cosmetic ingredients, pharmaceuticals, and environmental pollutants), which may influence their bioactivity and safety [81]. This approach moves beyond compositional analysis to functional prediction, particularly regarding metabolite transformation pathways that mediate host–microbe interactions. These predictions provide a valuable starting point for hypothesis generation, and their integration with experimental metabolomic and microbiological approaches represents a promising direction for future research.

### 5.2. AI-Assisted Identification of Microbiome Signatures Associated with Skin Health

If SkinBug illustrates what the skin microbiome can do, a complementary question is whether and how these functional capabilities relate to the host’s physiological state. This question requires a shift from functional prediction to phenotype association. Carrieri et al. collected skin microbiome samples from the lower extremities of Canadian women and integrated these data with physiological indicators, such as skin water content, pH, and electrical conductivity [82,83]. Machine learning models, including Random Forest, XGBoost, and LightGBM, were applied to investigate the association between skin microbiome composition and skin status. The study found that a higher abundance of *Lactobacillus* was associated with improved skin hydration, whereas an increased abundance of the genus *Bergeyella* was linked to dry skin and an elevated risk of dermatitis. These associations suggest that microbial composition alone can serve as a predictive proxy for skin physiological status, although the underlying mechanisms—particularly which microbial metabolites mediate these effects—remain to be experimentally validated.

Building upon this, recent studies have begun to directly integrate microbiome and metabolomic data to enhance predictive accuracy and uncover mechanistic links. For example, Guo et al. integrated skin microbiome and metabolomic data using Random Forest models to predict phenotypic skin aging [84]. The younger-looking group exhibited greater microbial diversity, with Stenotrophomonas maltophilia enriched and positively associated with youthful skin characteristics. Specifically, models constructed using microbiome, metabolome, and combined multi-omics data retained 46, 205, and 133 predictive features, respectively. The microbiome-based model achieved an area under the receiver operating characteristic curve (AUC) of 0.72, whereas the metabolome-based model reached an AUC of 0.89. Integrating both datasets further improved predictive performance, with the combined model achieving the highest AUC of 0.90.

In addition to enhancing predictive power through multi-omics integration, machine learning can also uncover metabolic mechanisms when accounting for potential confounding factors. For instance, age influences both skin microbiome composition and physiological status. To mitigate this, researchers developed an age-adjusted machine learning (AAML) framework and applied it to facial microbiome data from 595 Korean women [85]. Park et al. found that *Corynebacterium propinquum* was associated with improved skin tone in middle-aged women—an association not detected in whole-age analyses. Subsequent in vitro functional analysis linked *C. propinquum* to resveratrol biosynthesis, and LC-QTOF analysis confirmed the production of resveratrol by this species. Experimental assays showed that resveratrol reduced melanin secretion, suggesting a plausible mechanistic pathway blinking the ML-identified microbial signature to host skin phenotypes. Although the physiological relevance of this pathway in human skin requires further in vivo validation, these findings illustrate that AI models, when combined with targeted experimental approaches, can provide valuable mechanistic hypotheses rather than merely identifying statistical associations.

Collectively, these applications illustrate AI’s primary current roles in skin microecology: directly predicting microbial functional potential, and identifying phenotype associations that provide a bridge toward metabolic mechanisms.

### 5.3. Limitations and Future Perspectives

The applications described above illustrate the potential of AI to decode the skin microecosystem, but they also raise fundamental questions about the reliability, interpretability, and biological relevance of AI-generated predictions. These concerns are not unique to skin microbiome research; similar challenges have been documented across biomedical AI applications. Small datasets can increase the risk of overfitting, while inadequate separation of training, validation, and test sets may lead to data leakage and overly optimistic performance estimates. In skin microbiome research, these challenges are compounded by several domain-specific factors. First, the low biomass of skin samples introduces substantial technical noise, which can obscure true biological signals and reduce model reproducibility across studies. Second, the pronounced inter-individual variability of the skin microbiome means that models trained on one population may perform poorly on another. Third, the temporal dynamics of the skin metabolome–microbiome network are rarely captured by static, cross-sectional datasets, limiting the ability of current models to predict dynamic ecological transitions. In addition, biological confounders such as age can simultaneously influence microbial composition and skin phenotypes, potentially leading machine learning models to identify associations that reflect shared covariation rather than direct biological relationships. Therefore, appropriate control of confounding factors should be incorporated into model development and interpretation.

Similar challenges arise in the application of AI to skin microbiome prediction. For example, Carrieri et al. reported larger prediction errors when a model trained on data from Canadian women was applied to women in England. The authors suggested that differences in age, skin hydration, microbiome composition, and sequencing depth and coverage could contribute to this decline in predictive performance. A similar decline in model performance has been observed in other skin-related machine learning studies. For example, a machine learning model for identifying mitochondrial-related biomarkers in atopic dermatitis achieved an AUC of 1.000 in the training cohort, which decreased to 0.810 in an external validation cohort [88]. These findings illustrate that models developed using a single cohort may not generalize readily to populations with different demographic, biological, or technical characteristics, highlighting the importance of external validation across diverse cohorts [82].

Beyond predictive performance and generalizability, the interpretation of model outputs also requires caution. Carrieri et al. used SHAP to identify bacterial genera that contributed to model predictions. However, the authors noted that these genera might have causal biological roles or simply act as non-causal markers [82]. Model interpretability can therefore help prioritize microbial signatures for further investigation, but it does not, on its own, establish causal relationships or reveal the mechanisms through which microorganisms influence host physiology. This distinction is particularly important in the context of the skin metabolome–microbiome network, where correlation between microbial taxa and metabolite levels does not necessarily imply that the microbes produce or consume those metabolites. Such interpretations should be considered in the context of the model’s predictive reliability, and proposed mechanisms require validation through targeted experimental studies.

This distinction is also important when AI is used to predict microbial metabolic functions. For example, SkinBug predicts potential skin microbiome-mediated metabolic reactions and links these predictions to candidate enzymes, microbial species, and skin sites. However, such computational predictions primarily indicate metabolic potential and should not be regarded as direct evidence that the predicted reactions occur in vivo. Therefore, AI-based functional predictions are valuable for generating hypotheses and prioritizing candidate microorganism–metabolite interactions, but experimental validation remains necessary to establish their biological relevance and underlying mechanisms [81].

One potential strategy to improve the biological interpretation and reliability of AI-based predictions is to integrate multiple layers of omics data. In skin microecology, combining microbiome data with metabolomic and other molecular information may provide a more comprehensive view of the relationships between microbial composition, metabolic activity, and host responses. However, the integration of heterogeneous omics datasets also presents substantial challenges. For example, a multi-omic study of skin wound healing integrating single-cell transcriptomic, proteomic, and extracellular matrix ultrastructural data found that individual transcript and protein levels showed relatively limited correspondence, with an average correlation below 0.5, whereas greater consistency was observed at the pathway level. These findings illustrate that different omics layers cannot necessarily be directly aligned and that their computational integration requires careful biological interpretation [89].

Beyond multi-omics integration, several methodological advances may help address the reliability and generalizability challenges discussed above. Federated learning, which enables model training across distributed datasets without centralizing sensitive data, offers a promising approach to overcome the sample size limitations that constrain many skin microbiome studies [86]. Transfer learning, in which models pre-trained on large-scale datasets are fine-tuned for specific skin microecological contexts, may improve performance in data-scarce settings [87]. In addition, explainable AI (XAI) methods, when combined with mechanistic experiments, can help distinguish causal microbial signatures from non-causal associations and guide targeted validation studies [90].

Finally, a major gap in the current literature is the limited number of AI models trained directly on paired microbiome–metabolome datasets. Future work should prioritize the generation of such datasets and the development of multi-omics integration models that can simultaneously learn from microbial composition, metabolic profiles, and host phenotypes. Such models would enable the direct prediction of metabolite-mediated host–microbe interactions, moving beyond the current reliance on microbial composition as a proxy for metabolic function. Future AI applications in skin microecology should therefore move toward larger, more diverse longitudinal cohorts, rigorous external validation, explicit control over biological and technical confounders, and the integration of microbiome, metabolome, host molecular, clinical, and environmental data. AI-derived associations and functional predictions should primarily be regarded as hypothesis-generating until their biological relevance and causal mechanisms are confirmed through targeted experimental validation.

## 6. Conclusions

The skin is a multi-layered ecosystem where structure establishes the physical canvas, microbes populate ecological niches, and metabolites serve as the chemical dialogue binding them together. This multilevel framework reveals that microecological balance is not a fixed compositional ratio but a dynamic chemical equilibrium, maintained through the continuous reciprocal exchange of metabolites between the host and its resident microbiota. The principal contribution of this review lies in integrating these traditionally separated structural, microbial, and metabolic dimensions into a unified framework. Unlike previous reviews that have addressed the skin microbiome, metabolome, or AI applications in isolation, this review establishes an explicit mechanistic link among them: skin architecture generates spatially heterogeneous metabolic landscapes, microbial communities adapt to and reshape these landscapes, and AI provides the computational means to decode the resulting metabolite–microbe–host network. The complexity of these interactions poses substantial challenges for traditional analytical approaches. While artificial intelligence enables a progressive deepening of understanding—from predicting microbial functional potential to decoding the microbiome as a carrier of biological information—the distinction between association and mechanistic inference remains a central challenge. AI-generated predictions require experimental validation to establish causal relationships. Looking forward, longitudinal studies, protocol standardization, and mechanistic validation remain critical priorities for translating the chemical language of the skin into personalized skin health management.

## Figures and Tables

**Table 1:** Summary of AI-enabled approaches and methodological frameworks for decoding skin microecology.

Approach	Biological Target/ Mechanism	Stage of Development	Main Applications	Relevance to Metabolite- Microbiome Axis	Key References	Advantages	Limitations/Challenges
AI-driven microbial metabolic function prediction (SkinBug)	Prediction of microbial enzymes and biotransformation pathways from skin microbial pan-genomes	Experimental computational	Prediction of microbial metabolic reactions, candidate enzymes, responsible species, and skin sites	Direct: predicts microbial capacity to produce or transform metabolites and xenobiotics	[81]	Moves from taxonomic composition to functional potential; enables hypothesis generation	Predicts metabolic potential rather than in vivo activity; requires metabolomic and experimental validation
ML-based microbiome-phenotype association	Associations between microbial composition and skin physiological phenotypes	Experimental	Prediction of skin hydration, pH-related status, dryness, and other physiological characteristics	Indirect: identifies microbial signatures associated with host phenotypes; metabolite mediators remain inferred	[82,83]	Identifies predictive microbial signatures; links microbiome composition with measurable skin phenotypes	Associations do not establish causality; population dependence; limited generalizability
ML-based microbiome–metabolome integration	Joint modeling of microbial and metabolic features associated with skin phenotypes	Experimental	Skin-aging prediction; multi-omics biomarker identification	Direct: Simultaneously incorporates microbiome and metabolome data to identify phenotype-associated signatures	[84]	Improves predictive performance; Captures complementary microbial and metabolic information	Limited paired datasets; high dimensionality; requires independent validation
Confounder-adjusted ML and experimental validation (AAML)	Identification of age-adjusted microbial signatures and microbe–metabolite–host pathways	Experimental	Identification of skin phenotype-associated microbes and validation of metabolic mechanisms	Direct and mechanistic: links *C. propinquum* → resveratrol → reduced melanin secretion	[85]	Controls biological confounding; combines ML prediction with metabolomic and experimental validation	Context/population specificity; requires targeted experimental validation
Federated and transfer learning	Distributed model training and knowledge transfer across datasets/ populations	Emerging/methodological	Increasing effective sample size; improving generalizability in data-scarce settings	Methodological: may facilitate modeling of distributed microbiome–metabolome datasets but does not itself generate metabolite evidence	[86,87]	Supports multi-cohort learning; may reduce data-sharing barriers and data-scarcity problems	Heterogeneous datasets, domain shifts, technical standardization, and limited validation in skin microecology
Explainable AI (XAI)	Interpretation of model predictions and identification of influential microbial/metabolic features	Emerging/methodological	Feature attribution, biomarker prioritization, hypothesis generation	Methodological: helps prioritize candidate microbe–metabolite relationships for mechanistic validation	[82]	Improves interpretability and guides targeted validation	Feature importance does not establish causality; interpretation depends on model reliability

## Data Availability

No new data were created or analyzed in this study. Data sharing is not applicable to this article.
